# Hydrogen sulfide improves postischemic neoangiogenesis in the hind limb of cystathionine‐*β*‐synthase mutant mice via PPAR‐*γ*/VEGF axis

**DOI:** 10.14814/phy2.13858

**Published:** 2018-09-02

**Authors:** Avisek Majumder, Mahavir Singh, Akash K. George, Jyotirmaya Behera, Neetu Tyagi, Suresh C. Tyagi

**Affiliations:** ^1^ Department of Physiology University of Louisville School of Medicine Louisville Kentucky 40202 USA; ^2^ Department of Biochemistry and Molecular Genetics University of Louisville School of Medicine Louisville Kentucky 40202 USA

**Keywords:** Angiogenesis, hydrogen sulfide, stress response

## Abstract

Neoangiogenesis is a fundamental process which helps to meet energy requirements, tissue growth, and wound healing. Although previous studies showed that Peroxisome proliferator‐activated receptor (PPAR‐*γ*) regulates neoangiogenesis via upregulation of vascular endothelial growth factor (VEGF), and both VEGF and PPAR‐*γ* expressions were inhibited during hyperhomocysteinemic (HHcy), whether these two processes could trigger pathological effects in skeletal muscle via compromising neoangiogenesis has not been studied yet. Unfortunately, there are no treatment options available to date for ameliorating HHcy‐mediated neoangiogenic defects. Hydrogen sulfide (H_2_S) is a novel gasotransmitter that can induce PPAR‐*γ* levels. However, patients with cystathionine‐*β*‐synthase (CBS) mutation(s) cannot produce a sufficient amount of H_2_S. We hypothesized that exogenous supplementation of H_2_S might improve HHcy‐mediated poor neoangiogenesis via the PPAR‐*γ*/VEGF axis. To examine this, we created a hind limb femoral artery ligation (FAL) in *CBS* ^*+/−*^ mouse model and treated them with GYY4137 (a long‐acting H_2_S donor compound) for 21 days. To evaluate neoangiogenesis, we used barium sulfate angiography and laser Doppler blood flow measurements in the ischemic hind limbs of experimental mice post‐FAL to assess blood flow. Proteins and mRNAs levels were studied by Western blots and qPCR analyses. HIF1‐*α*, VEGF, PPAR‐*γ* and p‐eNOS expressions were attenuated in skeletal muscle of CBS ^+/−^ mice after 21 days of FAL in comparison to wild‐type (WT) mice, that were improved via GYY4137 treatment. We also found that the collateral vessel density and blood flow were significantly reduced in post‐FAL CBS ^*+/−*^ mice compared to WT mice and these effects were ameliorated by GYY4137. Moreover, we found that plasma nitrite levels were decreased in post‐FAL CBS ^+/−^ mice compared to WT mice, which were mitigated by GYY4137 supplementation. These results suggest that HHcy can inhibit neoangiogenesis via antagonizing the angiogenic signal pathways encompassing PPAR‐*γ*/VEGF axis and that GYY4137 could serve as a potential therapeutic to alleviate the harmful metabolic effects of HHcy conditions.

## Introduction

Homocysteine (Hcy) has been studied extensively for over 30 years for its unique involvement in an increasing number of human diseases (Hankey and Eikelboom 1999; Narayanan et al. 2014; Stipanuk and Ueki 2011). The Hcy level is controlled by two major processes: around 50% of Hcy enters the transsulfuration pathway to produce cysteine, and the other half is remethylated back to methionine (Met) via the folate 1‐carbon cycle (Cascella et al. 2015; Veeranki and Tyagi 2013). Hcy is generally present in blood in four different forms: approximately 1% as free thiol, 70–80% as a disulfide‐bound to plasma proteins and the remaining 20–30% as a homodimer or heterodimer with other thiols (Hankey and Eikelboom 1999). Normal total plasma Hcy concentration in our body ranges from 5 to 10 *μ*mol/L; however, in a diseased condition, such as in hyperhomocysteinemia (HHcy), plasma total Hcy levels are increased (>15 *μ*mol/L) (Lehotsky et al. 2016). HHcy can be classified as moderate (15–30 *μ*mol/L), intermediate (30–100 *μ*mol/L), and severe (>100 *μ*mol/L) (Lehotsky et al. 2016). Notable, there are four ways people can develop HHcy: (1) consumption of a Met‐rich protein diet; (2) vitamin B_12_/folate deficiency; (3) presence of heterozygous/homozygous status for cystathionine‐*β* synthase (CBS^+/−^/CBS^−/−^); and (4) obstruction of renal clearance (Sen et al. 2010). Apart from these factors, genetic variants in Hcy metabolism enzymes such as 677C>T and 1298A>C in *the MTHFR* gene can also lead to HHcy (Brustolin et al. 2010; Iqbal et al. 2009; Kharb et al. 2016; Verhoef et al. 2005). There are also several other drivers like age, sex, physical activity, alcohol intake, certain medications, and different disease conditions (such as type 2‐diabetes) that can also modulate the functioning Met cycle leading to an increased total Hcy concentration in the blood (Diakoumopoulou et al. 2005). Hyperhomocysteinemia (HHcy) has been associated with severe skeletal muscle dysfunction (Brustolin et al. 2010; Kanwar et al. 1976; Kolling et al. 2013; Majumder et al. 2017; Miller et al. 2000; Valentino et al. 2010; Veeranki et al. 2015; Voskoboeva et al. 2018; Zoccolella et al. 2008), but the precise mechanism(s) is still unknown. Previous studies found that HHcy causes endothelial cell (EC) injury (Wang et al. 1997), inhibition of EC proliferation (Zou et al. 2011), reduction of bioavailability of vasoregulatory mediators (nitric oxide and endothelin) (Upchurch et al. 1997), and induction of oxidative/ER‐stress (Singh et al. 2018; Tyagi et al. 2005; Werstuck et al. 2001). How HHcy reduces neovascularization in the skeletal muscle is not precisely known.

The growth of new blood vessels from the preexisting vascular network is known as neoangiogenesis. When oxygen supply is low in a tissue or organ, it activates hypoxia‐inducible factor 1 (HIF1*α*), which sends a signal to nearest blood vessel, activating endothelial nitric oxide synthase (eNOS) and producing NO needed for vasodilation. Vascular endothelial growth factor (VEGF) increases permeability and separates pericytes leading to the degradation of the basement membrane, thereby activating metalloproteases such as MMP‐2 and 9 (Carmeliet and Jain 2011). These changes lead to EC proliferation and a concomitant migration in order to form new blood vessels (Duran et al. 2017). In recent years, it has been shown that Peroxisome proliferator‐activated receptor (PPAR‐*γ*) might be involved in neoangiogenesis via growth factors and cytokines that in turn stimulate migration, proliferation, and survival of these ECs (Biscetti et al. 2008).

PPAR‐*γ* belongs to the nuclear hormone receptor superfamily, and when specific ligands bind to the ligand‐binding domain of PPAR‐*γ*, a conformational change releases the bound corepressors (Chawla et al. 1994; Tontonoz et al. 1995). This allows coactivators like PGC‐1*α* and other coactivators to be recruited to the PPAR‐*γ* responsive genes’ promoters, thereby promoting the PPAR‐*γ*‐mediated transcription (Costa et al. 2010; Laha et al. 2018; Murphy and Holder 2000). HHcy reduces PPAR‐*γ* expression in ECs (Mishra et al. 2010). Studies have shown that PPAR‐*γ* could regulate neoangiogenesis via upregulating VEGF (Biscetti et al. 2008) and that can further activate eNOS (Kroll and Waltenberger 1998). Although, previous works showed that HHcy impaired neoangiogenic growth in muscle via reduction of HIF1*α* and VEGF levels, whether PPAR‐*γ* plays any role in this process had not been studied (Veeranki et al. 2014).

Hydrogen sulfide (H_2_S) is increasingly being recognized as an important signaling molecule in cardiovascular and nervous systems via its ability to neutralize a variety of reactive oxygen species (ROS) (Kimura et al. 2010; Mironov et al. 2017; Yang et al. 2015), as well as via increased cellular glutathione levels through activation of gamma‐glutamylcysteine synthetase, and reduction of the disulfide bonds (Calvert et al. 2010; Elsey et al. 2010; Fiorucci et al. 2006; Gadalla and Snyder 2010; George et al. 2018; Kimura 2010; Predmore and Lefer 2010; Szabo 2007; Wang 2003). Cystathionine *γ*‐lyase (CSE) and CBS are the main H_2_S‐generating enzymes, producing H_2_S from Hcy in the transsulfuration pathway. Patients with CBS deficiency tend to produce a lesser amount of H_2_S (Beard and Bearden 2011; Kozich et al. 2016); suggesting that these patients are likely more prone to oxidative stress‐mediated damages due to excessive production of Hcy (Szabo 2007). A study revealed that endogenous H_2_S could induce mRNA and protein expression of PPAR‐*γ* (Cai et al. 2016), indicating that exogenous H_2_S supplementation could be employed as a beneficial strategy to improve neoangiogenesis defect in HHcy patients. Hence, the purpose of our study was to answer the following questions: (i) Does HHcy inhibit neoangiogenesis via downregulation of angiogenic signals like HIF1*α* and VEGF in the postfemoral artery ligation (FAL) hind limb of CBS^+/−^ mice? (ii) Does HHcy inhibit PPAR‐*γ* expression which can further downregulate VEGF/eNOS signaling in the post‐FAL hind limb of CBS^+/−^ mice? And finally, (iii) does GYY4137 treatment improve neoangiogenesis via PPAR‐*γ*/VEGF axis after 21 days of FAL in the hind limb of experimental CBS^+/−^ mice?

CBS is one of the key enzymes in the transsulfuration pathway, and heterozygous CBS deficiency (CBS+/−) has proved to be a useful model for analyzing the effects of mild to a severe endogenous elevation in the levels of Hcy (Familtseva et al. 2014; Nandi and Mishra 2017; Narayanan et al. 2013; Tyagi et al. 2011, 2012; Watanabe et al. 1995; Winchester et al. 2018; Yang et al. 2018). Hence, in this study, we used CBS^+/−^ mouse model to dissect the effect(s) of HHcy on neoangiogenesis in the skeletal muscle and evaluate whether exogenous administration of GYY4137 (an H_2_S donor) could improve this effect(s). Our results indicate that H_2_S could be developed as a potential therapeutic agent to treat the neoangiogenic defects in skeletal muscle wherein HHcy is linked with a barrage of metabolic dysfunctions.

## Materials and Methods

### Animal maintenance, genotyping, and diet protocol

Male WT (C57BL/6J) and CBS^+/−^ (B6.129P2‐Cbstm1Unc/J 002853) mice were purchased from the Jackson Laboratory (Bar Harbor, ME). All animals were ∼8–10 weeks‐old and maintained in 12:12 h light–dark cycle with regular mouse chow diet in the animal facility of the University of Louisville. All animal protocols and care were carried out according to the guidelines of National Institute of Health (NIH Pub. No. 86–23, revised 1985) and were approved by the Institutional Animal Care and Use Committee (IACUC) of the University of Louisville, KY.

**Figure 1:**
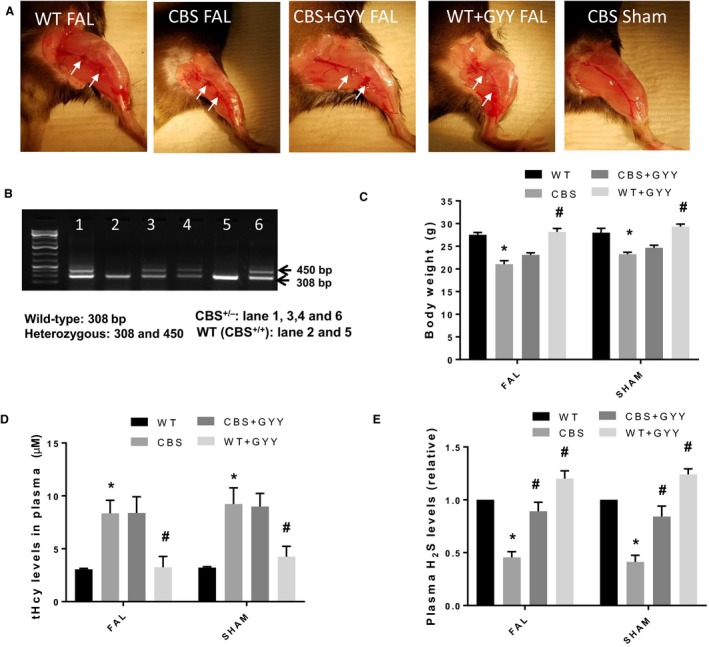
Phenotypic and genotypic correlations between cystathionine‐*β*‐synthase (CBS ^+/−^) and wild‐type (WT) mice groups. (A) Hind limb images after 21 days of GYY4137 treatments. (B) Genotyping for CBS ^+/−^ and WT mice. (C) Body weight measurements of experimental mice. (D) tHcy measurements from the plasma of experimental mice. (E) H_2_S measurements from the plasma of experimental mice. Data are shown as Mean ± SEM and mice number (*n*) = 4, statistical difference *P < 0.05 versus WT and ^#^ *P* < 0.05 versus CBS. (tHcy = total homocysteine, FAL = femoral artery ligation).

After purchasing mice were cross‐bred, yielding around 10% CBS^−/−^, 60% CBS^+/−^, and 25% CBS^+/+^. For genotyping, tail samples were collected, and DNA was isolated using DNeasy Blood & Tissue Kits (Qiagen, Germantown, MD). Genotypic analysis was performed using PCR by targeted disruption of the CBS gene at loci (representative images from each group of post‐FAL mice are shown in Fig. 1A and genotyping in Fig. 1B). CBS^+/−^ heterozygote mice produced two bands (450 and 308 bp), while wild‐type (CBS^+/+^) mice represented only one band (308 bp).

Animals were divided into four experimental groups: (1) Wild‐type C57BJ/L6 mice (**WT**), (2) CBS^+/−^heterozygous mice (**CBS**), (3) GYY4137‐supplemented CBS^+/−^ (**CBS+GYY**), and (4) GYY4137‐supplemented wild‐type mice (**WT+GYY**). A dose of 0.25 mg intraperitoneal injection was administered for mouse of GYY4137/kg body weight every day for a total 21 days after FAL surgery and while the WT mice were given 0.9% normal saline (vehicle control) (John et al. 2017).

### Femoral artery ligation

To create hypoxic condition, FAL (unilateral) was performed under intraperitoneal pentobarbital sodium (50 mg/kg) anesthesia as previously described (Beard and Bearden 2011; Kozich et al. 2016). Briefly, after separation of the femoral artery from the vein and nerve, it was ligated using 6‐0 silk suture at proximal and distal places (keeping same distance in all animals). We used separate mice groups [WT, CBS, CBS+GYY, and WT+GYY] as sham control where we passed the suture underneath the femoral artery but was not ligated. The skin was sutured using 6‐0 silk thread. After the skin closure, betadine was applied. After recovery, laser Doppler blood perfusion was carried out to confirm the induction of ischemia.

### Laser Doppler tissue perfusion imaging and flowmetry

MoorLDI (Moor Instruments) was used to measure tissue perfusion intensity and blood flow rates as described (Bhargava et al. 2014).

### Barium angiograms

To determine neoangiogenesis barium sulfate angiography was performed in mice as described (Givvimani et al. 2011). In brief, after pentobarbital anesthesia mice were infused with barium sulfate (0.1 g/mL) in 50 mmol/L Tris‐buffer (pH 5.0) at a constant flow (∼1 mL/min) and pressure with a syringe pump through the common carotid artery. Heparin (20 U/mL) was used along with barium sulfate to visualize the nascent neoangiogenesis. Angiograms were captured using the Carestream whole animal X‐ray imaging system (Carestream Molecular Imaging, Woodbridge, CT) as a previously described method (Machens et al. 2006) and the vessel density was quantified using VesSeg tool (Institute for Signal Processing, University of Luebeck, Lübeck, Germany).

### Reagents and antibodies

All reagents and chemicals were ordered from Sigma–Aldrich or available elsewhere but with highest grade.

The antibodies for HIF1*α* (ab51608), VEGF (ab51745), and eNOS (ab66127) were ordered from Abcam (Cambridge, USA). Whereas PPAR‐*γ* (sc‐7273), p‐eNOS Ser 1177 (sc‐12972), rabbit anti‐mouse (sc‐358914), mouse anti‐rabbit (sc‐2357), and mouse anti‐goat (sc‐2354) were from Santa Cruz Biotechnology (Dallas, TX). The antibody for GAPDH (MAB374) was from EMD Millipore (Burlington, MA), and used for Western blots analyses as per the manufacturers’ protocols.

### Western Blotting

Protein expressions were assessed by Western blots as described (John et al. 2017). Briefly, at the time of sacrifice, gastrocnemius muscle from the ischemic leg of each mouse was quickly removed, snap‐frozen, and stored at –80°C until further use. Protein from samples was extracted by homogenizing in the ice‐cold RIPA buffer (Boston BioProducts, Worcester, MA) containing 1 mmol/L Phenylmethylsulfonyl fluoride (Sigma, Saint Louis, MO), and 1% protease inhibitors cocktail (Sigma) and sonicated employing the Sonifier 450 (Branson Ultrasonics, Danbury, CT). The homogenates were centrifuged 17,400*g* for 20 min at 4°C, and the supernatants were quickly stored at –80°C until further use. The protein contents were estimated by the Bradford assay. Equal amounts of proteins (50 *μ*g) were resolved on SDS‐PAGE (8%, 10%, 12%) and then transferred to polyvinylidene difluoride (PVDF) membranes. The respective blots were incubated with primary and secondary antibodies before visualizing them using the ECL Luminata Forte (Millipore, Temecula, CA) in a Bio‐Rad ChemiDoc system. The intensities of the bands were normalized to the housekeeping GAPDH for all the proteins examined. The quantification was performed using Image Lab™ Software (Bio‐Rad, Hercules, CA).

### Reverse transcription and real‐time quantitative PCR

Total RNA was extracted from muscle samples using a Trizol method as described (Rio et al. 2010). Then, RNA quality was determined by NanoDrop ND‐1000, and RNA with high purity (260/280~2.00 and 260/230~2.00) was used for q‐PCR analysis. Reverse transcription was performed according to manufacturer's protocol using high‐capacity cDNA RT kit from Applied Biosystems (Foster City, USA) for the primer sequences listed in Table 1. For RT‐qPCR, SYBR green‐based kit was used to measure the relative expression of each mRNA specific primers. Briefly, three steps cycling protocol was performed using 20 ng of cDNA template in a 20 *μ*L reaction volume under the following conditions: denaturation at 95°C for 15 min followed by 40 cycles of 94°C for 15 s, 55°C for 30 s, and 70°C for 34 s in which fluorescence was acquired and detected by Roche LightCycler^®^ 96 Real‐Time PCR System (Roche Diagnostics, IN). Following RT‐qPCR, analysis of melt curve was performed to validate the specific generation of the expected PCR product. GAPDH was used as an endogenous control (Quanta Biosciences, Beverly, MA).

**Table 1:** List of primers used for RT‐qPCR experiments

Genes	Forward primers	Reverse primers
HIF1*α*	5′‐TCAAGTCAGCAACGTGGAAG‐3′	5′‐TATCGAGGCTGTGTCGACTG‐3′
VEGF	5′‐CAGGCTGCTGTAACGATGAA‐3′	5′‐CAATTTGGCTCCTCCTACCA‐3′
PPAR‐*γ*	5′‐TTTTCAAGGGTGCCAGTTTC‐3′	5′‐AATCCTTGGCCCTCTGAGAT‐3′
NOS3	5′‐GACCCTCACCGCTACAACAT‐3′	5′‐TCTGGCCTTCTGCTCATTTT‐3′

### Total plasma Homocysteine, H_2_S, and nitrite measurement

Blood samples were collected in tubes containing a 1/10 volume of 3.8% sodium citrate from each mouse by cardiac puncture after euthanasia. Then, plasma was isolated by centrifugation at 2500*g* for 15 min at 4°C. Total plasma Hcy concentrations were measured in samples using the homocysteine assay kit (Crystal Chem, USA) as per manufacturer's instructions.

Plasma H_2_S was measured as a previously described method from our laboratory (Kundu et al. 2015).

Nitrite from the plasma was measured by a Griess reagent [0.1% *N*‐(1‐naphthyl) ethylenediamine dihydrochloride, 1% sulfanilamide, and 2.5% phosphoric acid] and using sodium nitrite (0.01–100 *μ*g) as a standard as described previously (Kalani et al. 2014).

### Statistics

All values are expressed as mean ± SEM. The interaction between groups was determined by one‐way or two‐way ANOVA, including a Tukey's post hoc analysis when significant interactions were observed. The threshold for significance was set at *P* < 0.05, and total number of mice (*n*) = 4–5 was subjected to experimentation from each group. For statistical analyses, GraphPad Prism (Ver 7, GraphPad Software) was used.

## Results

The phenotypic feature and genotype of WT and CBS^+/−^ mice are depicted in Figure 1A and B, respectively. In this study, we noticed that CBS mice had significantly lower body weights in comparison to WT mice; however, we did not see any difference in body weights between CBS versus WT after GYY4137 treatment for 21 days (Fig. 1C). We observed CBS mice had significantly higher levels of plasma tHcy compared to WT mice, and GYY4137‐supplemented CBS mice also had similarly higher plasma tHcy levels as that of the CBS mice (Fig. 1D). After 21 days of GYY4137 treatment, we wanted to examine the plasma H_2_S concentrations in the experimental mice. Results showed that plasma H_2_S levels were significantly lower in the untreated CBS mice compared to that of the untreated WT mice as expected; however, after administration of GYY4137 for 21 days we did notice that plasma H_2_S levels were significantly elevated in both the CBS and WT mice (Fig. 1E).

**Figure 2:**
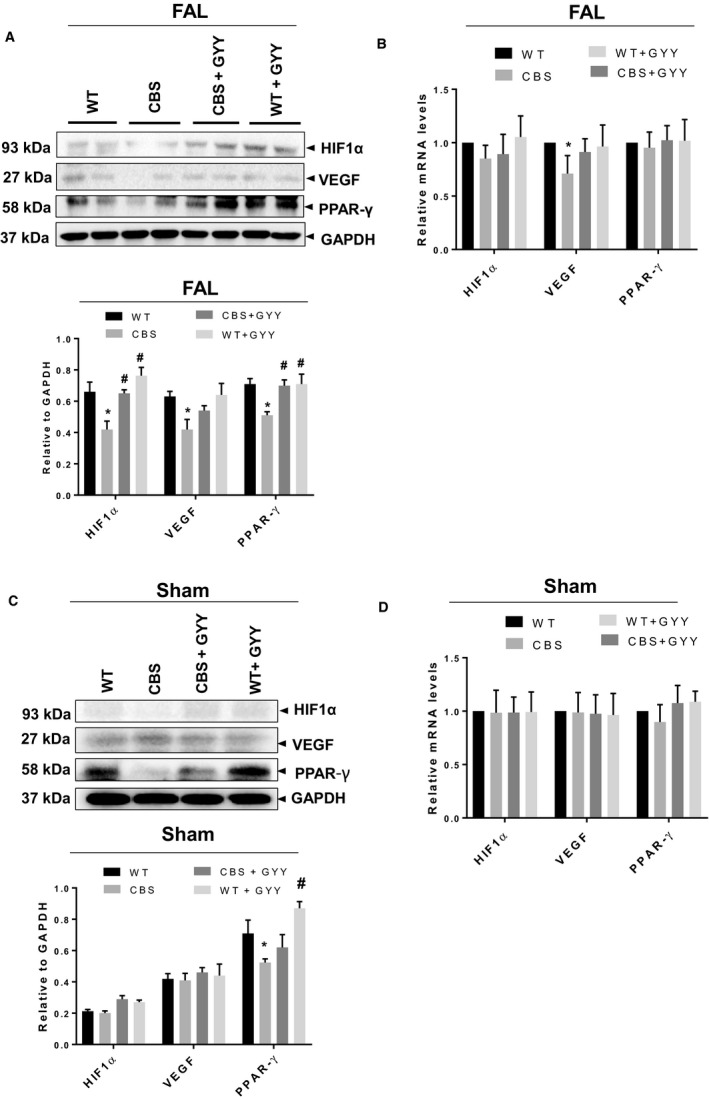
(A and B) Effect of GYY4137 on the improvement of neoangiogenic signals in skeletal muscle of post‐FAL cystathionine‐*β*‐synthase (CBS ^+/−^) mice. (A) Western blots analysis showing protein expressions: HIF1‐*α*, VEGF, and PPAR‐*γ* in the top panel and densitometric analysis of Western blots images are shown in the bottom panel. (B) mRNA expression for HIF1‐*α*, VEGF, and PPAR‐*γ* (log transformed). Data are shown as Mean ± SEM and mice number (*n*) = 4, statistical difference *P < 0.05 versus wild‐type (WT) and ^#^ *P* < 0.05 versus CBS (FAL = femoral artery ligation). (C and D) Effect of GYY4137 on angiogenic signals in skeletal muscle in sham CBS ^+/−^ and WT mice. (C) Western blots analysis showing protein expressions: HIF1‐*α*, VEGF, and PPAR‐*γ* in the top panel and densitometric analysis of Western blots images are shown in the bottom panel. (D) mRNA expression for HIF1‐*α*, VEGF, and PPAR‐*γ* (log transformed). Data are shown as Mean ± SEM and mice number (*n*) = 4, statistical difference *P < 0.05 versus WT and ^#^ *P* < 0.05 versus CBS.

As a marker of a hypoxia induction in post‐FAL hind limb, we measured the HIF1*α* levels by Western blotting. We found that HIF1*α* expression was higher in FAL mice compared to sham mice (Fig. 2A–D). However, when we examined the HIF1*α* levels between each group of FAL mice, we noticed that HIF1*α* levels were downregulated in post‐FAL CBS mice in comparison to WT mice, and GYY4137 treatment was found to mitigate this effect (Fig. 2A). To confirm mRNA expression of HIF1*α*, we did qPCR analysis, and it did not show any significant decrease in mRNA levels of HIF1*α* in post‐FAL CBS mice compared to post‐FAL WT (*P* = 0.4824), and GYY4137 treatment could not improve the mRNA levels in post‐FAL CBS mice (*P* = 0.9781) (Fig. 2B). We found that the protein expressions of VEGF and PPAR‐*γ* were reduced in post‐FAL CBS mice as compared to post‐FAL WT mice, whereas this effect was improved after GYY4137 administration (Fig. 2A). Additionally, in the qPCR analysis, we found mRNA expression of VEGF was significantly reduced in the post‐FAL CBS mice compared to post‐FAL WT mice (*P* = 0.0365); however, this effect was not improved upon GYY4137 administration (*P* = 0.2139). We did not notice any significant change in mRNA expression of PPAR‐*γ* among the four experimental groups (Fig. 2B). Besides, we did not find any difference in proteins and mRNA levels for HIF1*α* and VEGF among individual groups of sham mice as shown in Figure 2C–D. However, we did notice that the expression of PPAR‐*γ* was reduced in sham CBS mice compared to sham WT mice and that GYY4137 supplementation could not mitigate this effect. The observed reduction of PPAR‐*γ* mRNA level in sham CBS mice, compared to sham WT mice, was not statistically significant (*P* = 0.7423). Similarly, we did not observe, any significant improvement in mRNA expression for PPAR‐*γ* in sham CBS mice after GYY4237 treatment (*P* = 0.3549) (Fig. 2D).

**Figure 3:**
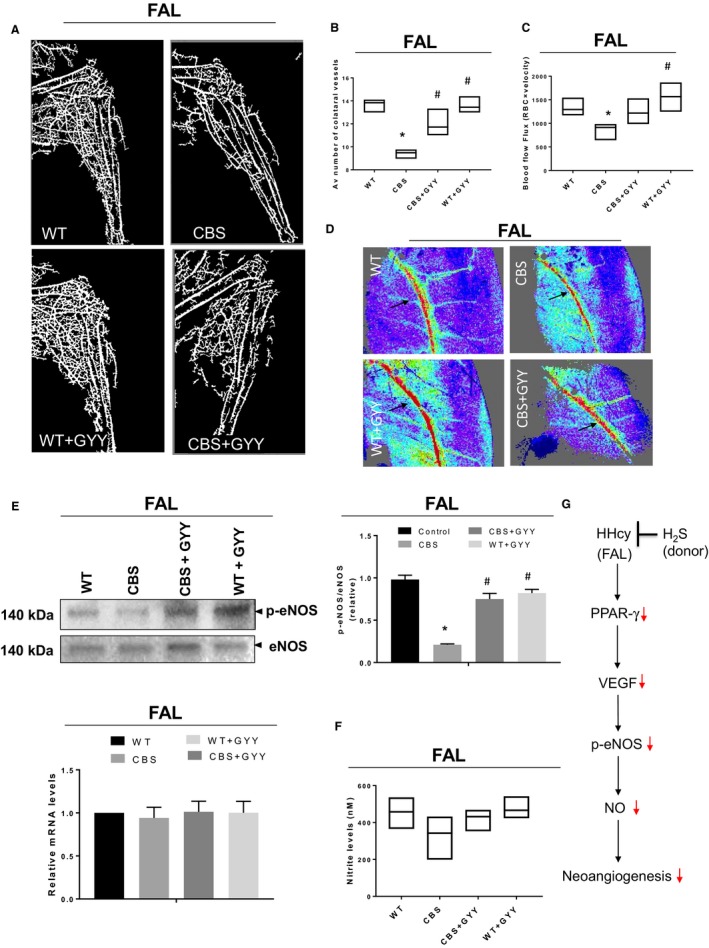
(A–D) GYY4137 supplementation improves neoangiogenic profile in the hind limb muscle of mice after 21 days of FAL in cystathionine‐*β*‐synthase (CBS ^+/−^) mice. (A) Barium angiogram images showing vascular density in the hind limb of skeletal muscle. (B) Quantitation of collateral vessel numbers in hind limb post‐FAL. (C) Blood flow rate measurements in hind limb post‐FAL. (D) Laser Doppler perfusion imaging showing the intensity of limb perfusion on 21 days after FAL. Data are shown as Mean ± SEM and mice number (*n*) = 4, statistical difference *P < 0.05 versus wild‐type (WT) and ^#^ *P* < 0.05 versus CBS (FAL = femoral artery ligation). (**E–G)** GYY4137 supplementation activates eNOS‐phosphorylation and plasma nitrite levels in CBS ^+/−^ mice. (E) Western blots analysis of protein expressions: p‐eNOS and eNOS in the top panel and densitometric analysis of p‐eNOS/eNOS ratio from Western blots images are shown in the right panel, and mRNA expression of NOS3 is shown in bottom panel. (F) Nitrite levels in plasma of post‐FAL mice measured by Griess assay. (G) Proposed model of this study is outlined. Data are shown as Mean ± SEM and mice number (*n*) = 4, statistical difference *P < 0.05 versus WT and ^#^ *P* < 0.05 versus CBS and ^#^ *P* < 0.05 versus CBS (FAL = femoral artery ligation).

Finally, we measured the vessel density employing barium sulfate angiography after 21 days of FAL surgery. We found that total collateral vessels’ number was significantly less in the post‐FAL CBS mice in comparison to post‐FAL WT mice, and this effect was further significantly improved upon GYY4137 treatment (Fig. 3A–B). Besides, we found blood flow in the hind limb after 21 days of FAL was reduced considerably in CBS mice compared to WT mice. It was improved by GYY4137 treatment as could be seen in Figure 3C–D. We did not notice any difference in the mRNA expression levels of NOS3; however, we observed that the p‐eNOS levels were reduced in the post‐FAL CBS mice as compared to post‐FAL WT mice, and interestingly, this effect was improved via GYY4137 treatment (Fig. 3E). The changes in the plasma nitrite levels in experimental mice were also monitored as the marker of nitric oxide (NO) production. Although not statistically significant, our findings revealed a reduction of plasma nitrite levels in post‐FAL CBS mice as compared to post‐FAL WT mice (*P* = 0.1050) with the GYY4137 administration improving this effect (*P* = 0.2923) (Fig. 3F). Based upon above findings, we have proposed a model that we firmly believe in application of H_2_S as a potential therapeutic intervention in treating neoangiogenic defects in skeletal muscle due HHcy (Fig. 3F).

## Discussion

Previous reports suggested several mechanisms for detrimental outcomes during the HHcy condition in different tissue types, including oxidative stress (Racek et al. 2005; Tyagi et al. 2005), protein homocysteinylation (Jakubowski 1999; Jakubowski et al. 2000), hypo/hypermethylation (Jiang et al. 2007; Narayanan et al. 2014; Pushpakumar et al. 2015; Yi et al. 2000), and endoplasmic reticulum stress (Perna et al. 2003). However, it is important to note that most of these observations are from in vitro studies using a supra‐physiological concentration of Hcy (~1 mmol/L) unlike as seen in HHcy patients. There are many in vivo models to study neoangiogenesis, either by passing a flexible wire or by applying a laser or an electrical current; however, none of these are relevant to clinical settings (Carmeliet et al. 1998; Lindner et al. 1993; Rosen et al. 2001). In this study, we used the FAL model by employing a genetically engineered mouse model (CBS^+/−^) mimicking HHcy conditions as seen in HHcy patients for the purpose of studying the postischemic neoangiogenesis phenomenon during HHcy. Our results add to the growing body of evidence that HHcy is associated with defective neoangiogenesis as shown in other in vivo experiments (Tan et al. 2006; Tawfik et al. 2013). Earlier, studies also showed that high Hcy (HHcy) has a profound inhibitory effect on EC proliferation and their migration (Cai et al. 2007; Li et al. 2006; Ozsvari et al. 2010; Papapetropoulos et al. 2009; Tan et al. 2006).

Neoangiogenesis is a natural process during chronic regional ischemia, which requires EC proliferation, migration, differentiation, and survival to form new blood vessels in order to compensate the hypoxic environment (Norton and Popel 2016). VEGF is a prototypical angiogenic cytokine that plays a vital role in this process and has been widely studied (Carmeliet 2000; Isner and Asahara 1999; Liu et al. 2000). A previous report involving hind limb ischemia in CBS^+/−^ mice showed no difference in VEGF levels after seven days of ischemia (Bosch‐Marce et al. 2005). However, they did notice a significant reduction in capillary density in the CBS mice compared to WT mice. Interestingly, our results demonstrate a significant decrease in HIF1*α* and VEGF expression after 21 days of FAL, suggesting that lower expressions of postischemic HIF‐1*α* may be responsible for delayed induction of VEGF in CBS mice compared to that of WT mice. The present study also explored mechanistic role of HHcy for the reduction of VEGF through PPAR‐*γ*‐dependent pathway. A study demonstrated that NaHS (H_2_S donor) treatment significantly improved capillary density and angiographic scores resulting in enhancement of blood flow in the ischemic hind limb (Wang et al. 2010). Similarly, we also noticed that exogenous administration of GYY4137 (H_2_S donor) could successfully mitigate the HHcy‐mediated neoangiogenic defects in skeletal muscle of CBS^+/−^ mice (Fig. 3).

H_2_S has been studied extensively for its salubrious effects in the cardiovascular system demonstrating profound vasodilatation, vascular protection, homeostatic regulation of blood pressure, and many others (Calvert et al. 2010; Elsey et al. 2010; Gadalla and Snyder 2010; Predmore and Lefer 2010; Szabo 2007). A previous study using chicken chorioallantoic membrane model revealed that H_2_S increased the length and complexity of the vascular network (Papapetropoulos et al. 2009). Similarly, in this study, we also noticed that exogenous supplementation of GYY4137 could improve collateral vessels’ density after 21 days of FAL in the CBS mice. In agreement with our study, Moore and colleagues were able to show that intraperitoneal administration of NaHS (an H_2_S donor) induced neovascularization in an in vivo mouse model using a Matrigel plug assay (Cai et al. 2007). A previous report showed that genetic deletion/silencing of CSE (another H_2_S‐producing enzyme) in the endothelium, reduced migration and sprouting of ECs in vitro, where in VEGF played a critical mediator (Papapetropoulos et al. 2009). In this present study, we observed that PPAR‐*γ* and VEGF expressions were significantly downregulated in CBS mice compared to WT mice. We also demonstrated that these effects were mitigated via GYY4137 administration. This suggests that most likely VEGF is regulated via the PPAR‐*γ*‐dependent pathway further corroborating Biscetti et al. (2008) findings wherein they clearly showed that activation of PPAR‐*γ* led to endothelial tube formation and induction of VEGF in ECs. Similarly, other investigators revealed that inhibiting PDE activity by H_2_S induces PPAR‐*γ* protein and mRNA expressions (Cai et al. 2016).

NO is also an endogenous gasotransmitter that, like H_2_S, is involved in vasorelaxation and stimulation of angiogenesis (Mustafa et al. 2009; Szabo 2010). HHcy was also found to quench NO (a vasodilator) by the formation of peroxynitrite anion (ONOO^−^) and uncoupling of eNOS, further reducing the bioavailability of NO (Dimmeler et al. 1999; Morbidelli et al. 2003; Topal et al. 2004). Similarly, we noticed nitrite levels and phosphorylation of eNOS were found to be reduced in CBS mice in comparison to WT mice. eNOS is known to produce NO during neoangiogenesis via VEGF (Kroll and Waltenberger 1998), and thus it appears that impaired angiogenesis in HHcy could be due to the reduction of NO availability. In this work, we demonstrated that nitrite levels and eNOS activation were reduced in the CBS mice compared to WT mice, and their levels could be mitigated via GYY4137 treatment. These findings are also highly consistent with previous reports where H_2_S was shown to stimulate Akt in ECs leading to the induction of eNOS through phosphorylation of Ser1177 (activation site) and a parallel dephosphorylation Thr495 (inhibitory site) (Coletta et al. 2012; Osipov et al. 2009).

In conclusion, our work embodies the proangiogenic role of H_2_S molecule. Pertinent findings from this study have been elaborated in a flowchart/model (Fig. 3G) highlighting the plausible intracellular signaling pathway of how H_2_S could mitigate the neoangiogenic defects during HHcy. We opine that additional pathways might be at work during neoangiogenesis (Majumder et al. 2018); however, further investigation needs to be undertaken involving similar but not identical scenarios wherein muscle dysfunction is the outcome of metabolic derangement. In brief, H_2_S does hold potential ramifications toward developing it as a clinically relevant therapeutic option for chronic conditions that are implicated in a host of inflammatory and cellular stress injury including the apparent defect in the neoangiogenesis (Majumder et al. 2018).

## Conflicts of Interest

No conflicts of interest, financial or otherwise, are declared by the authors.
